# Perceptions of key informants on the provision of cervical cancer prevention and control programme in Uganda: implication for cervical cancer policy

**DOI:** 10.1186/s12889-020-09482-y

**Published:** 2020-09-14

**Authors:** James Henry Obol, Reema Harrison, Sophia Lin, Mark James Obwolo, Robyn Richmond

**Affiliations:** 1 University of New South Wales School of Public Health and Community Medicine, Kensington, NSW 2033 Australia; 2 Gulu University, Faculty of Medicine, P. O Box 166, Gulu, Uganda

**Keywords:** Key informants, Perceptions, Cervical cancer, Policy, Uganda

## Abstract

**Background:**

Uganda has one of the highest burdens of cervical cancer globally. In 2010 the Ugandan Ministry of Health launched the Strategic Plan for Cervical Cancer Prevention and Control with the hope of developing cervical cancer policy in Uganda. This study explored the beliefs of senior key informants in Uganda about cervical cancer prevention, the control programme, and the relevance of cervical cancer policy.

**Methods:**

We conducted 15 key informant interviews with participants from six organisations across Northern and Central Uganda. Participants were drawn from district local government health departments, St. Mary’s Hospital Lacor, Uganda Nurses and Midwifery Council, non-governmental organisations (NGOs) and Ministry of Health in Kampala, Uganda. The interview recordings were transcribed and analysed using thematic analysis.

**Results:**

Seven themes emerged relating to the cervical cancer prevention and control programmes in Uganda: (1) policy frameworks for cervical cancer, (2) operationalising cervical cancer prevention and control, (3) financial allocation and alignment, (4) human resources and capability, (5) essential supplies and vaccines, (6) administrative data and resource distribution, and (7) cervical cancer services.

**Conclusions:**

The key informants perceive that the lack of a cervical cancer policy in Uganda is hindering cervical cancer prevention and control programmes. Therefore, the Ministry of Health and stakeholders need to work together in coming up with an effective policy framework that will accelerate efforts towards cervical cancer prevention and control in Uganda.

## Background

In Africa, cervical cancer incidence among women is second after breast cancer and is the number one cause of cancer mortality among women [1]. Screening programmes for women aged 35 to 64 years of age using conventional cytology at 3 to 5 year intervals has led to a decline of about 80% in invasive cervical cancer incidence among women screened in high income countries [2]. However, cervical cancer remains a substantial global health challenge in low- and middle-income countries (LMICs). In 2018, cervical cancer incidence worldwide was estimated at 569,847 cases, with sub-Saharan Africa (SSA) contributing about 20% to the total global incidence [3]. In the same year, the global mortality due to cervical cancer was estimated at 311,365 deaths, with SSA accounting for around 25% of the global cervical cancer death [3].

With the exception of South Africa, SSA countries lack national and regional cervical cancer policies [4]. It is well established that the presence of cervical cancer policy contributes to effective cervical cancer prevention and control activities. For example, the introduction of cervical cancer policy in Australia (1991) and United Kingdom (UK) (1988) was associated with a re-screening rate at 39 months of 74% in both countries [5]. Similarly, the cervical cancer incidence fell by 33% both in the UK and Australia while mortality also fell by 36% in both countries over a period of 6 years [5]. In the absence of cervical cancer policy, SSA countries face substantial challenges in the prioritisation, coordination and implementation of cervical cancer prevention and controls programmes. Challenges include: lack of organised cervical cancer screening services [6]; lack of or inadequate healthcare infrastructure and supplies [7]; lack of trained medical oncologists due to training costs [8]; lack of integration of cervical cancer screening in existing Human immunodeficiency virus/Acquired immune deficiency syndrome (HIV/AIDS) services [9]; and underfunding of healthcare expenditure among African countries [10]. Recent report of 2020 indicate that despite HPV vaccine being available since 2006, less than 30% of LMICs have introduced HPV vaccination in their countries compared with over 80% in developed countries [11]. The same can be said on cervical cancer screening in which only about 20% of women in LMICs who have ever been screened for cervical cancer compared with 60% of women in developed countries [12]. The World Health Organization recommends screening of women between the age of 30–49 years every 3 to 5 years and in countries where population based screening for cervical cancer has been implemented, there has been remarkable decline in mortality due to cervical cancer [13].

Most SSA countries have widespread poverty which acts as a barrier to accessing education that supports the development of women’s knowledge base regarding seeking cervical cancer services [14]. Generally, there is poor knowledge about cervical cancer and its risk factors in SSA countries even among educated women including health workers [14–17]. Furthermore, many women in SSA cannot access cervical cancer prevention and treatment services as they cannot afford to pay for either transport or medical fees [18]. In SSA, socio-cultural practices such as polygamous marriages, early age at sexual debut, high parity and underage marriages are common and these practices increases the risk of a woman developing cervical cancer [14, 19, 20]. Studies have documented cultural resistance to cervical cancer screening even where the services exist and these women normally present late when the cervical cancer is in advance stage [21, 22]. SSA countries have suffered from civil conflicts which have caused the destruction, looting and abandonment of the health facility by health workers [23] thereby rendering delivery of healthcare services including for cervical cancer impossible. During war and other disasters, population get displaced and lived in camps where vices such as prostitution, multiple marriages and cohabitation, rape and sexual promiscuity among men are common occurrences leading to transmission of HPV to women [24].

Global commitments and actions with indicators have sought, in recent years, to reduce the burden of cervical cancer. For example, the World Health Organization (WHO) 90–70-90 triple-intervention strategy aimed to achieve 90% HPV vaccination coverage, 70% women being screened at least twice in their lifetime and 90% of women having access to cervical precancer and cervical cancer treatment and palliative care services by 2030 [12, 25]. If the WHO triple-intervention strategy is implemented between 2020 and 2030, the combined intervention of HPV vaccination and cervical cancer screening would avert between 300,000–400,000 deaths from cervical cancer in LMICs [25]; global alliance for vaccine and immunisation (GAVI) commitment to support HPV vaccination in low income qualifying countries [26]; the 2015 world declaration to reduce premature death due to non-communicable diseases (NCDs) by one-third by 2030 as reflected in sustainable development goal (SDG) 3, target 3.4 [25]; the WHO 2017 position paper on HPV vaccines [27]; development of safe and effective HPV vaccines [28]; Global taskforce on expanded access to cancer care and control in developing countries [29]; and political declaration in 2011 on NCDs prevention and control during UN High-Level meeting [30].

In Uganda, cervical cancer has been rising at the rate of 1.8% per annum for a period of 20 years [31]. Furthermore, the majority of cervical cancer patients present at UCI for treatment with late stage disease [32]. Uganda is among the high burden countries with age-standardised cervical cancer incidence rate at 54.8 cases per 100,000 women per annum and age-standardised mortality rate of 40.5 deaths per 100,0000 women annually [33]. The incidence and mortality due cervical cancer in Uganda is similar to other SSA countries such as in Tanzania, where the age-standardised incidence rate for cervical cancer is at 59.1 cases per 100,000 women and age-standardised mortality rate for Tanzania is 42.7 deaths per 100,000 women [33]; and in Burundi with an age-standardised incidence rate at 57.4 cases per 100,000 women and age-standardised mortality rate due to cervical cancer is at 50.3 deaths per 100,000 women per year [33]. The 5-year relative survival for cervical cancer patient in Uganda is estimated to be 17.7% [34], in Kenya is 19.8% [35] and Malawi 2.9% [36].

In line with the global commitment to reduce on the high burden of cervical cancer, the Uganda government launched a Strategic Plan for Cervical Cancer Prevention and Control in 2010 [37]. One of the implementation modalities proposed was the development of a national policy on cervical cancer prevention and control in Uganda [37]. Yet since the launch of the Strategic Plan, no information regarding cervical cancer policy development in Uganda has been forthcoming. In 2015, the Uganda Ministry of Health introduced Human Papillomavirus (HPV) vaccination among girls aged 10–14 years or in primary 4 (grade 4) class as primary mean to prevent cervical cancer [38, 39]. The Uganda Ministry of Health has set a target of 80% of HPV vaccination coverage for girls aged 10–14 years [37]. Between 2015 and December 2017, the annual coverage of the HPV vaccine first dose was at 85% for the target girls. However, annual coverage of the HPV vaccine second dose within same period has been inadequate with only about 41% of target girls receiving the vaccine [39]. Furthermore, available evidence from 2006 to 2019 indicate that use of cervical cancer screening services among Ugandan women has remained low ranging from about 5–35% [40].

Despite the unveiling of the Strategic Plan, funding for cervical cancer prevention and control activities are reliant on donors or non-governmental organisations (NGOs), leading to variable coverage and provision occurring in a few selected areas [41]. The lack of universal coverage means that many households in Uganda have no access to prevention and control services.

To address the shortcoming associated with cervical cancer prevention and control in Uganda, there is need for cervical cancer policy in Uganda. This will provide a basis for the development and optimisation of sustainable cervical cancer prevention and control programmes and activities. Additionally, having a cervical cancer policy in place will address challenges such as acceptability, feasibility, adoption and appropriateness of the services [42].

As a precursor to policy development, this study was conducted to explore the perceptions of key informants at senior levels in Ugandan health policy and services regarding the current status of cervical cancer in Uganda and the factors affecting delivery of cervical cancer prevention and control programmes.

## Methods

### Design

This was a qualitative cross-sectional study of key informants conducted in Uganda during March and April 2019. Key informants interviews (KII) allowed participants to provide detailed personal insight [43].

### Sample

A diverse range of participants from northern and central Uganda were purposively selected for the KII because of their experience in implementing cervical cancer prevention and control programme. This research sought to gain insight from key informants regarding the cervical cancer prevention and control program in Uganda, therefore we sought conceptual depth as opposed to data saturation [44]. We sent invitation letters to 25 potential participants identified as key informants based on their roles in their organisation (Ministry of Health, Reproductive Health Uganda, Mariestope Uganda, District Health Offices, St. Mary’s Hospital Lacor, and Uganda Nurses and Midwives Council) requesting them to participate in the interview.

### Quality control procedures

We used a semi-structured interview guide with questions framed around the World Health Organization (WHO) Health System Six Building Blocks which are leadership/governance, health care financing, health workforce, medical products and technologies, information and research, and service delivery [45]. The interview guide enabled prompting and probing for clarification on emerging concepts [43]. The guide was piloted with two Ugandan senior health professionals and one professor of public health from University of New South Wales (UNSW) then refined based on their feedback. Interviews were conducted in English in each of the participants’ offices and audio recorded. Our interview questions were open-ended, and participants discussed the questions without any restriction but guided to keep focus to the topic under discussion. We used probes to obtained detailed information for responses. To improve the validity of our findings, the audio recording was played back at the end of each interview so that the participant can confirm, add or withdraw any part of a statement they had made during the interview that did not reflect what was intended. Each interview lasted for approximately 30 min and was audio recorded for transcription.

### Data analysis

The audio recordings were transcribed verbatim and analysed using Nvivo version 12 software [46]. The analytical method was based on Framework Methods based on Ritchie and Lewis (2003) as described by Gale et al [47]. This method was selected due to its successful use with interview data from policymakers and managers in other healthcare settings, such as in the UK [48, 49]. The Framework Method enables researchers to explore qualitative data in the context of an analytic framework relevant to the research aims and questions, leading to themes and broader categories of findings. It therefore provides a structure within which a thematic content analysis can be undertaken, allowing a focused analysis to address policy-relevant research questions [47]. This form of thematic analysis offers a strategy for data reduction, in which the complex and large data can be reduced to draw out the key concepts, whilst highlighting idiosyncrasies and divergent cases [49]. This process offers a comprehensive exploration of the data. It enabled us to look beyond the content of the discussion to identify where, when, and why themes emerge; contextualising themes in the discussion which could reveal further detail.

Following transcription, line-by-line coding was conducted, identifying key words, phrases, and sentences, focusing on the themes that developed from the data. Coding was iterative and refinement of themes and subthemes evolved over the course of the analysis. A team-based approach to coding [50] was used to establish inter-rater reliability. Co-authors RH and JHO independently read transcripts and formulated the emerging coding frame. Then the research team met, discussed, and agreed on the coding framework to be used in the final data analysis. All transcripts were subjected to line by line coding within the coding framework. Themes were grouped under categories and given labels with references to the raw data. Discrepancies between the reviewers were discussed and themes refined until full agreement was reached [51]. This method of triangulation helps to confirm the observations, adds to the breadth of analysis as well as validate study results [52].

### Ethical considerations

This study was approved by University of New South Wales Human Research Ethics Committee (UNSW-HREC) number HC180508, Gulu University Research Ethics Committee (GU-REC) GUREC-090-18 and registered with the Uganda National Council for Science and Technology (UNCST) under number SS4839. All the heads of organisations whose staff participated in this study provided a written administrative clearance for us to interview their staff. All participants provided written informed consent before taking part in the interviews and all interviews were coded to preserve anonymity.

## Results

A total of 15 participants (8 males, 7 females) were interviewed from six organisations, across northern and central regions of Uganda. This included participants from district local government health department; St. Mary’s Hospital Lacor; Uganda Nurses and Midwives Council (UNMC); two non-government organisations (NGOs) and the Ministry of Health in Uganda. The participants included: national Senior Programme Officer (1), Medical Director from St. Mary’s Hospital Lacor, a Catholic Mission not for profit hospital, the largest hospital in the northern region (1), Programme Managers from NGOs supporting reproductive health (2), Midwives (2), Senior Nursing Officers from maternity (1), District Health Officers (DHO) (3), Assistant DHO in-charge of maternal and child health (3), Medical officer in charge of clinical services in a district (1) and chairperson of Uganda nurses and midwives’ council on education, training and enrolment committee (1).

Seven themes emerged underpinning cervical cancer prevention and control efforts in Uganda. These were: (1) policy frameworks for cervical cancer, (2) operationalising cervical cancer prevention and control, (3) financial allocation and alignment, (4) human resources and capability, (5) essential supplies and vaccines, (6) administrative data and resource distribution, and (7) cervical cancer services.

### Policy frameworks for cervical cancer

Almost two-thirds of the respondents (9/15) reported that they were not aware of a Uganda Ministry of Health cervical cancer policy, only of the national health policy that covered cervical cancer. There was a strong view that if there was a policy on cervical cancer prevention and control in Uganda, participants would know of it and refer to it in their day-to-day work. Although participants did not recall a policy on cervical cancer, they reported receiving an information pamphlet on the implementation of Human Papillomavirus (HPV) vaccination among adolescent girls. Most of the participants stated that having a cervical cancer policy is important to them while performing their duties as it would guide service planning and delivery to the population.

*KII (05) – “I am not aware of any policy on cervical cancer”.*

*KII (15) – “We have the national health policy and cervical cancer is one of the diseases covered”.*

Eight of the 15 respondents stated that cervical cancer is grouped under a broad category of non-communicable diseases at the district level. They considered that this grouping affected funding allocation for cervical cancer prevention and control activities in Uganda. Participants reported that there is a mismatch between funding priorities and population health needs in Uganda. For example, HIV/AIDS prevalence among women aged 15–49 years is 7.5% [53]. These women are at increased risk for cervical cancer but cannot access screening services due to the lack of government provision of the basic reagent such as acetic acid to the health facilities.

*KII (13) – “At policy level, the resource allocation to that sector is low”.*

*KII (03) – “We planned for some of these activities, but they are put under unfunded priority and so if an NGO comes to the district and they have the resources then they can come in otherwise to say this is a budget allocated quarterly or in a financial year is not there”.*

### Operationalising cervical cancer prevention and control

Most participants (13/15) talked about lack of central coordination for cervical cancer prevention and control activities both at the national and district levels. At the national level, most participants were unsure whether cervical cancer is positioned within reproductive health or non-communicable diseases. The positioning of cervical cancer was considered critical to inform the district health department so that they can know which office to engage in relation to cervical cancer activities. Similarly, absence of oversight of cervical cancer at the district level was problematic.

*KII (03) – “At the national level I think it is being coordinated by the reproductive health division of the Ministry of Health”.*

*KII (04) – “I think someone in charge of non-communicable diseases”.*

*KII (01) – “There is no focal person for cervical cancer but for Human Papillomavirus immunisation is under the cold chain person”.*

*KII (04) – “At the district here, I am the one responsible not for cervical cancer per se but sexual and reproductive health which cervical cancer is part of. It is one of the components of sexual and reproductive health. I am the district focal person”.*

All participants indicated that there is a process for supervision of cervical cancer services at the national level. Integrating all the diseases under non communicable diseases (NCDs) was discussed as an approach that, whilst maximising the available resources, came at the expense of quality due to the vast number of diseases under the NCD umbrella. The volume of other NCDs was discussed as leading to a lack of focus on cervical cancer. Two-thirds (10/15) suggested that to improve the quality of cervical cancer services, supervision should be specifically for cervical cancer.

*KII (15) – “We conduct integrated support supervision for NCDs, so we look at cancer and cervical cancer is one of them. We don’t reach all health facilities in the country. We sample a few because of the resource constrains”.*

*KII (13) – “Sometimes most of this supervision is integrated. As it is integrated, it may have some challenges in itself. It may not be very specific. Preferably, there is need for some of this supervision to be specific so that it can address the challenges in the affected communities”.*

National supervision for cervical cancer activities to the district was considered by many participants (11/15) not to be regular like that of diseases such as malaria, HIV and Tuberculosis (TB). Irregular supervision limited discussion about challenges faced by those trying to implement cervical cancer prevention, such as the low HPV-2 vaccination uptake, and how to overcome those challenges.

*KII (02) – “It is supposed to be on quarterly basis but sometimes you find in a year, it is done once, sometime twice but most time they take long”.*

Of the seven participants working at the district level, all but one believed that the current supervision was less supportive but more service delivery supervision. Six of the district level respondents indicated that supportive supervision needs guidelines for cervical cancer activities such as screening and treatment using cryotherapy. These services and guidelines were reported as unavailable. These six respondents also indicated that they were only conducting HPV vaccinations and reporting on the number of people who are vaccinated. Even among the four participants from NGOs that provided reproductive health services including cervical cancer, all four participants indicated that they do not receive supportive supervision from the district health department.

*KII (01) – “It is not a kind of supportive supervision. What we have is service delivery and when you do service delivery, you report on the people who have got the services. So, I think that can be an area of weakness”.*

*KII (08) – “We don’t receive supportive supervision, but we report to the district the activities carried out so that it is reported at the district level as activities carried within the district”.*

### Financial allocation and alignment

Two-thirds of the respondents (10/15) discussed lack of defined funding sources for cervical cancer activities and shared their frustration at the lack of funding. Participants described the impacts of lack of defined funding in terms of service delivery for cervical cancer at the national level as there is no budget for cervical cancer activities by Ministry of Health.

*KII (15) – “That is again tricky because, apart from those projects which partners were implementing mainly for cervical cancer screening and treatment of precancerous lesion, there is no specific money that is for cervical cancer. We have challenge of stock-out of supplies including nitrous oxide gas for treatment of precancerous lesions and also, other supplies for screening because there is no specific budget for cervical cancer. It is still a big challenge”.*

All the eight government employee participants indicated that funding to the district is fixed with specified items approved for expenditure. Participants further noted that cervical cancer is not identified as a priority disease by the Uganda government, despite the serious health risk posed. The district health departments rely on NGOs funding. This funding is inconsistent, and this has created challenges in the implementation of cervical cancer prevention and control activities by the district health department.

*KII (03) – “For us we don’t have specific budget that this is for this, but we have primary healthcare (PHC) fund and is from the Ministry of Health. The problem is that the PHC fund is just by name that the money is sent but it can’t do everything. It starts to cover outreaches, cleaning of compound, hiring support staff. At the end become a drop in the ocean”.*

*KII (02) – “Actually, in the past we used to have a partner call NUHITES, they used to support cervical cancer screening so much because each time we could organize camp, women would really come but this days, we have tried with these present partners we have on the ground, you know they come with their interest where they want to perform so they are not into cervical screening so much”.*

All government employee participants expressed frustration with the accounting system which does not allow them to reallocate funding to support activities that they deem important such as cervical cancer. Reallocating primary healthcare funding to support cervical cancer activities was discussed by these eight participants as a misappropriation of funds, which is a criminal offence and therefore a significant deterrent.

*KII (13) – “This funding which is there is always tag to a particular kind of activity and if it is not for cervical cancer, should you first borrow from antenatal and put it into cervical cancer activities. You will be forced to account. That is misappropriation of fund. So, the fund that is always sent you cannot reallocate it to other priority of the district”.*

Most participants (11/15) reported that there is lack of community advocacy for cervical cancer that leads to cervical cancer not being prioritised by the government. Participants believed that cervical cancer is an important emerging health problem but that the community is less aware of it than communicable diseases.

*KII (13) – “Let me take it from the perspective of non-communicable disease is taking little attention of the public”.*

### Human resources and capability

All participants stated that the implementation of cervical cancer activities were integrated within the existing health care delivery system in the district health facilities. At the health facility level, senior nursing officers and head of health facility are left to ensure that the services are provided in their facility.

*KII (14) – “We don’t have a standalone structure but use the existing structure which fall under the docket of someone in-charge of maternal and child health who is senior nursing officer”.*

Health facilities reported having a range of staff involved in cervical cancer activities with midwives identified as the central profession for cervical cancer screening. Participants further cited that midwives are preferred for training by NGOs so that when they are trained, they can both screen for cervical cancer and administer intrauterine device (IUD) contraceptive to women.

*KII (04) – “Basically, are the midwives. Maybe some nurses but not clinical officers. Not that they don’t know but basically sometimes this come as project, donor driven and most donors sometimes when they train, they say we want to train people so the first people they think of are normally midwives. For us in the lower health facilities screening is conducted by midwives because they are the people who have access to the maternity where screening is conducted and are more used to speculum. Clinical officers are more on outpatient sides, general cases”.*

Many participants (11/15) discussed the lack of training for those providing cervical cancer services. Furthermore, training was considered to be poorly organised, with a lack of involvement of the district health department in the training process. Only a few health workers are selected for training by partners with the hope that they return and mentor other staff.

*KII (13)* – *“Sometime a partner may come and take one or two people and train them on cervical cancer and they are to come and mentor the rest in the district”.*

All the three DHOs and three assistant DHOs stated that they were unable to organise training for other health workers because they do not have a district training manual to guide the training content. The majority of participants from districts (6/7) indicated that the NGOs that organised the training of health workers did not give the district health department a copy of the training manuals and other essential documents.

*KII (01) – “They didn’t hand over training manual”.*

### Essential supplies and vaccines

The supply of the HPV vaccine is done by the National Medical stores, this means that the vaccines’ supply is more guaranteed than other cervical cancer prevention and control equipment and commodities, which are currently not supplied by the National Medical stores.

Acetic acid, an essential component of cervical cancer screening using visual inspection with acetic acid (VIA), is not provided by Ministry of Health Uganda. All participants voiced their disapproval that the government did not supply acetic acid and that it has created a big gap in cervical cancer screening provision at the district level.

*KII (04)– “Incidentally, they don’t even give us something for cervical cancer, the acetic acid. It is not part of the essential supplies. Maybe what can be there is the speculum which is a fixed asset if it is provided once, it is in the health facility”.*

The central procurement process for medical supplies in Uganda and delivery by national medical stores was considered a key factor in creating both delays and inadequate supply.

*KII (01) – “Sometimes they are not delivered timely and this put an element of inadequacy Just as you found in some health facilities HPV vaccines were not there”.*

Despite the challenges surrounding delivery of essential commodities for cervical cancer prevention and control, participants reported that they have devised ways for managing shortages within their organisations. With 10 respondents indicating that they either reallocate supplies from one health facility to the one in short supply within the district or borrow from NGOs or neighbouring districts.

*KII (08) – “If it is not adequate or timely, we normally send an emergency requisition because it will not wait for the normal dispatch of items. Also, we try following it up with district or other organisation so that if they have, they avail us with it so that we continue providing the services”.*

*KII (05) – “We can relocate from one facility to another within the district”.*

*KII (13) – “We inform national medical store for supply or borrow from neighbouring districts if we have shortage”.*

### Administrative data and resource distribution

All the participants agreed that the health facilities use health management information systems (HMIS) in paper form to collect data. Despite the health facilities generating data on cervical cancer, participants reported that the data is not used to inform their planning, budgeting and decision making. Nine respondents indicated that health workers just compile and submit monthly reports as required. Without data on the burden of cervical cancer within the community, it is difficult to promote awareness of the issue with community members or government officials.

*KII (02) – “What I have seen mainly and this is one other thing which we discussed of recently that our health workers have not taken that seriously like not only on cervical cancer mainly but generally they have never taken data as something which they can come out with some decision out of it or data can make an informed decision”.*

A system of financing activities based on data generated from previous activities was proposed by participants as a strategy to enhance data utilisation. With generated data used to inform the work plan, budget and funding of the activity, they could more readily establish the population health risks and allocate appropriate funding.

*KII (05) – “For now, their plans are fixed but we think with result-based financing they can use it for planning outreaches and improving services because they have additional funding. Data on immunisation for HPV is very useful for planning and budgeting for outreaches by health facilities”.*

### Cervical cancer services

The range of cervical cancer prevention and control activities was considered inadequate according to 10 of the 15 respondents; cervical cancer activities are left to NGOs to implement and do not cover all districts. Lack of sustainability of cervical cancer screening services beyond the NGO project lifespan was identified as a key challenge. Furthermore, these respondents stated that there has been minimal attempt at scaling up of proven interventions after initial projects.

*KII (12) – “Cervical cancer prevention and control activities in Uganda is sporadic. I would not say something which is well organised. There are projects which comes and goes away, some campaign has been organised by development partners. Screening either VIA, Pap test or other methods, these have not been sustained in most facilities except when there is a specific project and when funding for the project ends, there is no real plan to scale it up.”*

Whilst the community was considered to be aware of the availability of HPV vaccines in the health facilities, nine respondents identified a lack of sufficient knowledge regarding why the vaccine is given to girls 10–14 years in the community and primary 4 class (grade 4) school girls. The lack of knowledge why government is immunising girls aged 10–14 years in the community or those in primary 4 class (grade 4) by the community has led to mistrust of the HPV vaccine, affecting vaccine uptake. Most participants (9/15) claimed that when health workers go for outreaches in schools, they vaccinate girls who are in primary 4 class (aged 10–14 years) and girls from other classes are left wondering.

*(KII 01) – “The community is aware about HPV vaccination but there is still knowledge gap. What I can say is that there is need for more information, health education, more sensitisation both to the community and these girls being given vaccination because if you go and give injection to a 10 year old girl in Primary level 4, the others in primary level 5 and 3 are always wondering what is this, why are they giving only that class, so there is need to be a lot of information”.*

Lack of community awareness about cervical cancer screening was also identified as a challenge, compounded by negative attitudes among some health workers who were not willing to screen women.

*(KII 04) – “I think you know we have other pressing issues. People think….., you know cancer is normally silence. There are diseases which normally come aggressive which people focus normally goes to like malaria, HIV, TB. You know where the clients are many at once, but cancer is something that is silence and yet is very deadly. Clients are really not well oriented that I have to go for routine screening especially the people at risk. Even if you go so what? You will go there, and they will say, mummy what do you want?”*

Almost all respondents (14/15) stated that patients with advanced cervical cancer face difficulties in accessing treatment in the region. These 14 respondents indicated that cervical cancer patients with advanced conditions are referred to the Uganda Cancer Institute (UCI) in Kampala, which is the only treatment facility for advanced cancer in Uganda. Most participants (10/15) said that lack of means of travelling and subsistence while at UCI, made most patients to return to their homes.

*KII (12) – “Cervical cancer patients come in different stages but unfortunately most of them come late. The one who come early can be treated surgically because we have the gynaecologist. The advanced cancer, we can make the diagnosis, confirm the diagnosis and we prefer to refer them. The problem is that most of our mothers are so poorer, they cannot even think of going to the only cancer institute in Uganda. So, most of them prefer to go back home and we don’t know what happen to them. Of course, eventually they die”.*

Cervical cancer patients are provided with a referral letter and left to access the next level of care themselves, with participants reporting that women were not followed up. A way to determine if a woman has gone for treatment and the treatment outcome was considered critical.

*KII (07) – “We have the referral forms which we give to the client to come to Gulu Regional Referral Hospital”.*

*KII (12) – “The capacity would be when they come back here for follow-up with the referral form then we can look at whether she had been treated. Often you see, this is a weak system. Referral system is still a problem. We are not even sure if they reach there. There is no means of checking. So, this area needs to be improved on. Mothers come from different areas and they go on their own because we don’t refer them using ambulance. When they reach there, we don’t know unless the referral centre give us feedback but there is no means may be until we use electronic then that would help but the paper work, they go with and give to the doctor and when they are discharge, they are not given back the referral paper and they don’t come back to referral facility but go to their homes. Because coming back to the referral facility needs some money and so it is a difficult process and it is not only for cervical cancer but for other conditions as well. So, the system needs to be improved upon”.*

## Discussion

In this study, seven core themes on cervical cancer prevention and control in Uganda emerged which are: 1) policy frameworks for cervical cancer, 2) operationalising cervical cancer prevention and control, 3) financial allocation and alignment, 4) human resources and capability, 5) essential supplies and vaccine, 6) administrative data and resource distribution, and 7) cervical cancer services. The policy frameworks for cervical cancer as a theme was prominent in the data and it underpinned the other themes as it determined allocation of resources and service delivery for cervical cancer prevention and control in Uganda. Given the competing health demands in Uganda, it is necessary to have a cervical cancer policy in place for cervical cancer to be prioritised by Government and health care workers. Research conducted in Kenya found that it was necessary to have a policy to support HPV vaccination and its integration into other healthcare delivery services [54]. In Nigeria, study has found that in the absent of cervical cancer policy, cervical cancer prevention programme will not be sustainable [55]. In India, cervical cancer policy was a necessary prerequisite to aligning resources and streamlining cervical cancer services delivery given competing health priorities [56].

In this study, operationalising cervical cancer prevention and control is necessary so that district health departments and NGOs are aware of the appropriate overseeing department. We contend that operationalising cervical cancer prevention and control may also ensure that representatives are available at the national and district levels for oversight and problem solving to ensure continuity of services. Nakisige highlighted the lack of coordinated effort from the Uganda Ministry of Health which had limited success [32]. Furthermore, the inclusion of cervical cancer under the NCD portfolio compromises the quality of cervical cancer services.

A lack of regular quarterly supervision for health workers compromised the prompt identification of barriers to cervical cancer service provision. Supervision effectively monitors the delivery of cervical cancer prevention and control services and enabled programme evaluation. In Malawi, it was noted that supervision was generally weak and was conducted on a quarterly basis only during the donor’s support [57].

Undependable and erratic cervical cancer funding was noted as one barrier to the provision of cervical cancer prevention and control activities. NGO funding, when available, is limited always for feasibility or demonstration project in few selected areas thereby limiting many Ugandan women from accessing vital preventive services for cervical cancer. Cervical cancer is not regarded as a priority disease and as such there is no budget to support implementation of activities related to prevention and controls. Other studies have reported lack of government funding and a high prevalence of NGO funding of cervical cancer programmes in Uganda, Kenya and Tanzania [41, 58, 59]. Through community advocacy, the participants of this study believed that cervical cancer would gain government attention and this will enable improvement in funding allocation to implement prevention and control services in the community.

In Uganda, cervical cancer annual mortality is alarming at 2275 death (27.2 deaths per 100,000 women) [60]. There is a high level of community awareness about cervical cancer [61–63]. However, for the community to participate in effective advocacy, they need to be mobilised so that they can campaign for better services. This requires the collection of complete and accurate data on the burden of cervical cancer so that community leaders can advocate for better funding.

In this study, we found that midwives were at the forefront of screening women for cervical cancer. However, the participants noted the limited involvement of district health departments in organising or supervising training. This casts doubt on the quality of the training as they are not aware of the training materials and competency of the trainers. A study in Guatemala found that many NGO VIA training programmes were not standardised and were a source of quality concerns among key informants [64]. Among the few midwives who were trained with the hope of providing mentorship to other midwives, there were also concerns about quality of the mentorship. Furthermore, there is an urgent need for a training manual to be provided to the district health departments to enable training for other midwives and nurses who could help in scaling up quality cervical cancer screening services in the various health centres within the district. Limited training had previously been noted as one of the health system barriers for effective implementation of cervical cancer screening programme [65].

The integration of cervical cancer prevention and control activities within the existing health care structure is good as indicated by our participants. This will enhance sustainability and optimal utilisation of scarce resources. However, this could make many staff that were not trained or poorly trained to conduct cervical cancer screening. A study has documented lack of trust by other health workers in the VIA screening results by health workers who were not trained as major obstacle in implementation of cervical cancer screening [66].

The VIA is currently recommended for cervical cancer screening in Uganda but is highly subjective and there is need for periodic refresher training and monitoring to ensure quality assurance is maintained by the service providers [67]. In the absence of leadership to offer supportive supervision of the screening programme, there is great risk to the quality of the cervical cancer screening services as there is no one to ensure that there is quality assurance and quality control in the service provided. Integrating cervical cancer screening into existing primary healthcare services without proper leadership leads to lack of accountability and inconsistency in service quality [65]. The Uganda Ministry of Health adopted VIA as the main screening method in 2010 [37]. However, we have shown that 10 years later, the Ministry of Health does not regularly provide acetic acid to the health facilities to facilitate this screening.

Our study indicates that there is need to strengthen the use of data generated from the health facilities by the health facility staff to promote informed planning and decision making. One possible option proposed by participants is to provide funding of cervical cancer activities based on the output data from the health facility. This is because output data can guide in planning and making informed decision on the resources required to provide community services. The use of data by the health workers will ensure quality improvement through data quality assessment which will enhance confidence in the generated data, and promote skills in data analysis by the health workers [68].

The present study shows that the range of cervical cancer prevention and control services are inadequate. Furthermore, the participants noted that where screening was initiated by NGOs, there is lack of sustainability or plan to scale up the services by government. Holme and colleagues argued that for cervical cancer programme to be sustained or scaled up, political will is required to make the necessary investment [69]. Mistrust by the community also was mentioned as another factor which hinders uptake of the current HPV vaccination programme. Participants noted that the community is not well educated about the targeted age group for the vaccination and this has created mistrust leading to low uptake of HPV-2 vaccination. Mistrust about HPV vaccine has been reported among East African immigrants in the US [70], African immigrant in England [71] and among Europeans [72].

Access to cervical cancer prevention and control services was reported to be a major challenge among women in Northern Uganda. The lack of community awareness about cervical cancer screening and negative attitudes by some health workers were cited as barriers to screening access. Lack of awareness in the community and negative attitudes by health workers were reported as a barrier to accessing cervical cancer prevention and control services [73]. Access of treatment by patients with advanced cervical cancer were cited as problematic since there is no capacity to provide treatment nor capacity to provide patients with transport within Northern Uganda. Patients with advanced cervical cancer are referred to UCI in Kampala. However, the lack of transport and money for living expenses while at UCI, leads many women to not access treatment. Barrier to accessing treatment related to transport cost have been reported in a study conducted in Zimbabwe [73]. Furthermore, there is a weak referral follow up system which is unable to track referred patients to the next level of care facilities. Earlier research has documented weak referral networks as one of the barriers to accessing treatment by cervical cancer patients [74].

### Limitations

Our study explored the perceptions of a diverse group of health workers at various levels working for government and NGOs in Uganda. Participants who were managers may have been reserved in their responses due to their positions and roles within their organisations. However, to ensure free discussions, participants were constantly assured that their identity will remain anonymous in all research output and this enables our participants to open-up during the discussion. Furthermore, the purposive selection of participants plus non-participation of some potential participants in the study could have introduce bias in the results observed.

## Conclusion

Our findings reveal several challenges faced in the provision of cervical cancer prevention and control in Uganda. These challenges are in part a result of the absence of a cervical cancer policy which has affected the organisation of cervical cancer prevention and control programmes in Uganda. There is an urgent need for the development of a cervical cancer policy in Uganda to streamline and help mobilise and direct resources to facilitate cervical cancer prevention and control activities.

## Data Availability

We included various quotations from datasets within this manuscript to show our study findings. However, we cannot make the complete datasets or the generated transcribed interviews which we analysed for this study available to the public. This is because of confidentiality agreement with our research participants since we had agreed to keep individuals’ identity anonymised in our research output.

## Abbreviations

AIDS: Acquired immune deficiency syndrome
DHO: District Health Officers
GU-REC: Gulu University Research Ethics Committee
HIV: Human immunodeficiency virus
HMIS: Health management information systems
HPV: Human Papillomavirus
IUD: Intrauterine device
KII: Key informants interviews
LMICs: Low- and middle-income countries
NCDs: non communicable diseases
NGOs: non-governmental organisations
PHC: Primary healthcare
SSA: sub-Saharan Africa
TB: Tuberculosis
UCI: Uganda Cancer Institute
UK: United Kingdom
UNCST: Uganda National Council for Science and Technology
UNSW: University of New South Wales
UNSW-HREC: University of New South Wales Human Research Ethics Committee
VIA: Visual inspection with acetic acid
WHO: World Health Organization
